# Whole-genome resequencing analyses of five pig breeds, including Korean wild and native, and three European origin breeds

**DOI:** 10.1093/dnares/dsv011

**Published:** 2015-06-27

**Authors:** Jung-Woo Choi, Won-Hyong Chung, Kyung-Tai Lee, Eun-Seok Cho, Si-Woo Lee, Bong-Hwan Choi, Sang-Heon Lee, Wonjun Lim, Dajeong Lim, Yun-Gyeong Lee, Joon-Ki Hong, Doo-Wan Kim, Hyeon-Jeong Jeon, Jiwoong Kim, Namshin Kim, Tae-Hun Kim

**Affiliations:** 1 Animal Genomics and Bioinformatics Division, National Institute of Animal Science, Rural Development Administration, Jeonju 565-851, Republic of Korea; 2 Korean Bioinformation Center, Korea Research Institute of Bioscience and Biotechnology, Daejeon 305-806, Republic of Korea; 3 Department of Bioinformatics, Korea University of Science and Technology, Daejeon 305-806, Republic of Korea; 4 Swine Division, National Institute of Animal Science, Rural Development Administration, Suwon 441-706, Republic of Korea

**Keywords:** whole-genome sequencing, Pig, SNP, signature of selection

## Abstract

Pigs have been one of the most important sources of meat for humans, and their productivity has been substantially improved by recent strong selection. Here, we present whole-genome resequencing analyses of 55 pigs of five breeds representing Korean native pigs, wild boar and three European origin breeds. 1,673.1 Gb of sequence reads were mapped to the Swine reference assembly, covering ∼99.2% of the reference genome, at an average of ∼11.7-fold coverage. We detected 20,123,573 single-nucleotide polymorphisms (SNPs), of which 25.5% were novel. We extracted 35,458 of non-synonymous SNPs in 9,904 genes, which may contribute to traits of interest. The whole SNP sets were further used to access the population structures of the breeds, using multiple methodologies, including phylogenetic, similarity matrix, and population structure analysis. They showed clear population clusters with respect to each breed. Furthermore, we scanned the whole genomes to identify signatures of selection throughout the genome. The result revealed several promising loci that might underlie economically important traits in pigs, such as the *CLDN1* and *TWIST1* genes. These discoveries provide useful genomic information for further study of the discrete genetic mechanisms associated with economically important traits in pigs.

## 1. Introduction

Domestication of pigs originated from local wild boar populations in Europe and Asia circa 10,000 yrs ago^1^ and occurred independently from wild boar subspecies in Europe and Asia.^2^ There are hundreds of domestic pig breeds available across the world, and pigs have served as one of the most important sources of animal protein for humans. Since the last century, traditional selective breeding has contributed significantly to the genetic improvement of economically important traits in pigs. For example, substantial improvements were achieved in pigs per sow per year (annually +0.25) and the feed conversion ratio (annually −0.009 to −0.070 kg of the dry matter intake per live mass gain kg) during recent decades.^3,4^ Furthermore, some pig breeds have been used as mammalian model animals for the biomedical research because of their physiological and anatomical similarities to humans, and for developing several swine lines, including highly inbred miniature pig breeds.^5,6^ However, most of the achievements were made in European origin breeds, whereas, there are fewer systematic genetic improvement programs for diverse Asian local breeds.

There are two types of Korean indigenous pigs currently registered with the worldwide Domestic Animal Diversity Information System of the Food and Agriculture Organization of the United Nations: Chookjin Chamdon and Jeju native pig.^7^ However, they are generally termed as the Korean native pig because of their same origin until the middle of the last century. Korean native pigs are known for their peculiar characteristics including higher intramuscular fat contents and chewy texture, which are appealing palatability factors to Korean pork consumers. However, they also have poorer productivities, such as lower growth rates and litters per sow per year than typical imported breeds, such as Duroc and Yorkshire.^8,9^ Since the beginning of the last century, the native breeds have been endangered, especially because of the extensive crossbreeding with economically promising imported breeds. To conserve the native genetic resource, recent efforts have been made by the National Institute of Animal Science (NIAS) in Korea to restore and manage the Korean native population.^10^ The Korean native pig is genetically more closely related to other Asian native pigs than to most European origin pigs.^11^ Kim et al.^12^ further showed that it might have a distinct genetic distance even from some Chinese native pig breeds.

The completion of the swine sequencing project has resulted in many single-nucleotide polymorphisms (SNPs) being identified throughout the genome.^1^ The recent advances in next-generation sequencing (NGS) technologies have led to further cataloguing of SNPs by resequencing of diverse pig breeds or multiple animals in comparison with the reference sequence assembly. Furthermore, the SNPs derived from NGS have been used successfully to dissect genomic characteristics of pigs. For example, Rubin et al. revealed significant signatures of selection in pig genomes by scanning whole genomes using SNPs.^13^ In addition, three Berkshire pigs were resequenced recently to explore their genetic relationship with 38 pig genomes obtained from the public database, providing useful information on the breed's origin and domestication.^14^

In this study, we present whole-genome sequencing analyses of 55 pigs of five breeds, including Korean wild boar, Korean native, Duroc, Landrace, and Yorkshire. Substantial numbers of SNPs were identified across the genome using the Swine reference assembly (*Sus scrofa* 10.2). SNP sets derived from the whole-genome sequencing were used to explore genomic characteristics among the diverse pig breeds and to detect genomic regions potentially implicated in the strong selective breeding applied to the pig populations in this study.

## 2. Materials and methods

### 2.1. Sampling and DNA extraction

We sequenced 55 animals from five pig breeds, including 10 Korean wild boars, 10 Korean native pigs (4 boars and 6 sows), 6 Duroc pigs (3 boars and 3 sows), 14 Landrace pigs (7 boars and 7 sows), and 15 Yorkshire pigs (7 boars and 8 sows) for this study (Table 1). The Korean native pigs were accessed at the Swine Science Division, NIAS, Rural Development Administration (RDA), Suwon; and the Subtropical Animal Experiment Station in NIAS, RDA, Jeju. The Duroc, Landrace, and Yorkshire pig samples were collected at the NIAS and Sunjin Co., Ltd in Korea. The Korean wild boars were captured in either Gyeonggi or Gyeongsangnam Provinces in South Korea, sampling five animals from each province, respectively. Genomic DNA was extracted from blood samples in EDTA using a Wizard Genomic DNA kit (Promega, Madison, WI, USA). The genomic DNA of the Korean wild boars was extracted from meat samples. The quality and quantity of the DNA were evaluated using a NanoDrop spectrophotometer (NanoDrop Technologies, USA) and gel electrophoresis in 1% agarose gel. The concentration of double-stranded DNA was checked using a Qubit dsDNA HS Assay (Invitrogen, USA). The National Institute of Animal Science's Institutional Animal Care and Use Committee reviewed and approved the study protocol and standard operating procedures (No. 2009-077, C-grade).

**Table 1.** Summary of sequencing results and SNPs from the five pig breeds including KWB, KNP, DUR, LAN, and YOR used in this study

Sample name	No. of sample	Raw_Reads	Mapped reads	A_Coverage^a^	Ave_Fold^b^
Korean wild boar	10	4,637,563,674	2,881,666,665	98.72%	11.21 X
Korean native pig	10	4,738,183,150	3,017,226,894	98.94%	11.74 X
Duroc	6	2,134,670,142	1,788,806,414	98.16%	11.60 X
Landrace	14	6,092,030,408	4,652,217,189	99.18%	12.93 X
Yorkshire	15	7,330,204,916	4,225,021,030	98.86%	10.96 X
Total	**55**	**24,932,652,290**	**16,564,938,192**	**99.18%**	**11.71 X**

^a^A_Coverage, assembly coverage calculated as the proportion of bases in the genome assembly that were covered by at least one read.

^b^Ave_Fold, average fold that was calculated as the average depth of coverage across the whole genome.

### 2.2. Library construction and sequencing

DNA libraries were constructed to have insert sizes of ∼300 bp, according to the manufacturer's instruction (Illumina, San Diego, CA, USA). The libraries were sequenced using the Illumina HiSeq 2000 platform (paired-end 101-bp reads) and Illumina GA IIx platform (paired-end 100-bp reads). We generated 24,932,652,290 reads (2,518 Gb) from 55 samples. Each sample was sequenced to have over >34 Gb initial reads to produce a minimum of 10-fold genomic coverage.

### 2.3. Mapping, SNP calling, and annotation

The sequenced reads were mapped to the Swine reference genome assembly (Sscrofa10.2) using Burrows Wheeler Aligner version 0.5.9 with default options.^15^ The genome sequence was downloaded from the UCSC genome browser (http://hgdownload.soe.ucsc.edu/goldenPath/susScr3/bigZips/susScr3.fa.gz). We also screened the unmapped reads and the reads that were aligned to unplaced scaffolds. Of the aligned reads, removal of polymerase chain reaction duplicates and re-synchronization of the mate information were performed using MarkDuplicates and FixMateInformation in the Picard software package version 1.48 (http://picard.sourceforge.net/), respectively. Local alignment around indels was performed to the duplication-removed reads using RealignerTargetCreator and IndelRealigner in the Genome Analysis Toolkit16 (GATK; version 1.0.5974). Furthermore, basepair quality scores were recalibrated using CountCovariates and TableRecalibration in the GATK.^16^ Multi-sample SNP genotyping was performed to identify SNPs using UnifiedGenotyper in GATK. To reduce the false discovery rate, the filtering steps followed these criteria: QUAL < 30.0, QD < 2.0, MQ < 40.0, FS > 60.0, HaplotypeScore > 13.0, MQRankSum < −12.5, and ReadPosRankSum < −8.0. All the filtered SNPs were annotated to belong to 12 functional categories (Table 2) using an SNP annotation tool, snpEff and snpSift version 3.6c,^17,18^ and the Ensembl *Sus scrofa* gene set version 75 (Sscrofa10.2.75). For the gene set, the canonical genes were applied to the annotation with the ‘-canon’ option in the snpEff program. Non-synonymous SNPs that had a SIFT score <0.05 were classified as potentially damaging variants. SIFT scores for the Sus scrofa gene set were downloaded from SIFT 4G (SIFT Databases for Genomes; http://sift-dna.org).

**Table 2.** Summary of all the detected SNPs identified from all of the five breeds used in this study

Fields	Total	KWB	KNP	DUR	LAN	YOR
Sample counts	55	10	10	6	14	15
SNP	20,123,573	13,973,333	9,592,404	6,625,918	10,872,881	11,032,246
Transition	13,352,480	9,266,133	6,304,688	4,351,967	7,166,396	7,259,773
Transversion	6,771,093	4,707,200	3,287,716	2,273,951	3,706,485	3,772,473
SNP categories^a^						
Synonymous coding	53,057	35,517	24,367	15,340	26,903	28,430
Non-synonymous coding	34,990	22,267	16,146	10,871	18,058	19,101
Start lost	26	15	15	9	14	15
Stop gained	393	230	189	92	189	214
Stop lost	49	39	25	22	31	28
Non-coding exon	96,102	60,277	46,017	26,706	51,131	51,305
UTR 5′	11,888	7,430	5,562	2,886	6,188	6,602
UTR 3′	68,432	46,001	31,269	21,670	35,645	36,640
Splice site acceptor	266	175	137	94	159	165
Splice site donor	275	193	133	96	161	160
Intron	4,955,607	3,445,188	2,337,787	1,602,182	2,660,083	2,690,109
Intergenic	14,902,488	10,356,001	7,130,757	4,945,950	8,074,319	8,199,477
Functional categories^b^						
Missense	35,065	22,321	16,186	10,902	18,103	19,144
Nonsense	393	230	189	92	189	214
Silent	53,057	35,517	24,367	15,340	26,903	28,430

UTR, untranslated region.

^a^SNP categories: categorized by the effects of SNPs.

^b^Functional categories: categorized by functional effects of coding SNPs.

### 2.4. Phylogeny and admixture analysis

To perform phylogenetic and admixture analyses, haplotypes of all the animals in this study were inferred by an imputation tool, BEAGLE, version 3.3.2.^19^ The phased haplotypes based on the SNPs were used to conduct phylogenetic analysis. The phylogenetic tree was generated using neighbour-joining tree estimation with pairwise distance, and Kimura's two-parameter distance method implemented in Analysis of Phylogenetic and Evolution (version 3.0.6) in R language.^20^ The tree was plotted using MEGA5,^21^ and the population structures were accessed by admixture analysis using the STRUCTURE version 2.3.4, which is based on the maximum likelihood method.^22^ We used different values of *K* (number of putative ancestral clusters of allelic similarity) and an admixture model with a correlated allele frequency to assign the *K* clusters.^23^ A 30,000 burn-in period of Chain Monte Carlo searches followed by 20,000 replicate runs were performed at each *K* from 2 to 5. The result was then plotted using DISTRUCT version 1.1.^24^ The pairwise similarity between each animal was computed by the number of the same SNP genotypes divided by the total number of SNPs. The order of the 55 animals for the STRUCTURE plot and the similarity matrix followed the same order used in the phylogenetic tree.

### 2.5. Identification of selective sweeps

To identify putative selective sweeps, we searched genomic regions with higher degrees of fixation, following the method by Rubin et al.^13,25^ The number of major and minor allele sequence reads were counted at each of the identified SNP in each breed. We filtered the SNP positions whose minor allele frequency was <0.05. We then scanned the genome using 50% overlapping windows of 150 kb in size, which was successfully adopted in the previous pig resequencing study.^25^ Among the 150 kb windows, we excluded windows with fewer than 10 of the number of heterozygous SNPs in each breed. Briefly, the Pooled heterozygosity (Hp) was initially computed by an equation: 2*sum_maj*sum_min/(*sum_maj*+ *sum_min*),^2^ where *sum_maj* and *sum_min* are the sum of major and minor allele frequency at the given 150 kb window, using the numbers of reference and alternative allele sequence reads observed in each of the breeds. The ZHp score was calculated from *Z* transformation of the Hp score as described by Rubin et al.^13,25^

### 2.6. Validation of SNPs by genotyping chip array

To validate the accuracy of SNP calling from whole-genome sequencing of five pig breeds, we genotyped five animals per each breed using the Illumina PorcineSNP60 v2 Genotyping BeadChip. Of the 64,232 SNP probes in the chip, we selected 44,903 probes that were successfully located on the autosomal and sex chromosomes. The reference allele of a chip SNP was set to the allele that is equal to the base at the reference genome, Sus scrofa 10.2. The genotype concordance was measured for each animal by the number of concordant genotypes between the SNP chip and the whole-genome sequencing derived SNPs divided by the total number of the SNP probes. The method was referred from GATK online documentation for VariantEval (http://gatkforums.broadinstitute.org/discussion/48/using-varianteval).

### 2.7. Data access

All SNPs detected in this study have been submitted to the NCBI dbSNP with the accession numbers: ss1754731760–ss1774855430.

## 3. Results and discussion

### 3.1. Sequencing and read mapping

We extracted genomic DNA from 55 pigs of five breeds including Korean wild boar (KWB), Korean native (KNP), Duroc (DUR), Landrace (LAN), and Yorkshire (YOR). Approximately 24.9 billion reads were generated by short-read sequencing technology including the Illumina GA IIx and HiSeq 2000 sequencing platforms. This equated to ∼2.52 terabases and 897-fold coverage compared with the expected genome size of the Swine reference genome (Sscrofa 10.2; size ∼2.8 Gb). To detect high-quality SNPs from all the samples, we sequenced at least 34 Gb for each of the samples. Of the 55 sequenced animals, the least amount of sequence reads was 12.1-fold for the ‘DUR5’ individual. After mapping the reads to the reference genome, we removed erroneous sequence reads that were caused by potential polymerase chain reaction duplication (17.5%). This yielded ∼16.6 billion reads (1,673.1 Gb), covering 99.2% of the reference assembly at an average of 11.7-fold coverage across the covered region (Table 1). There was no outstanding coverage bias observed on any specific chromosome within each of the animals.

### 3.2. SNP detection

**Figure 1.**
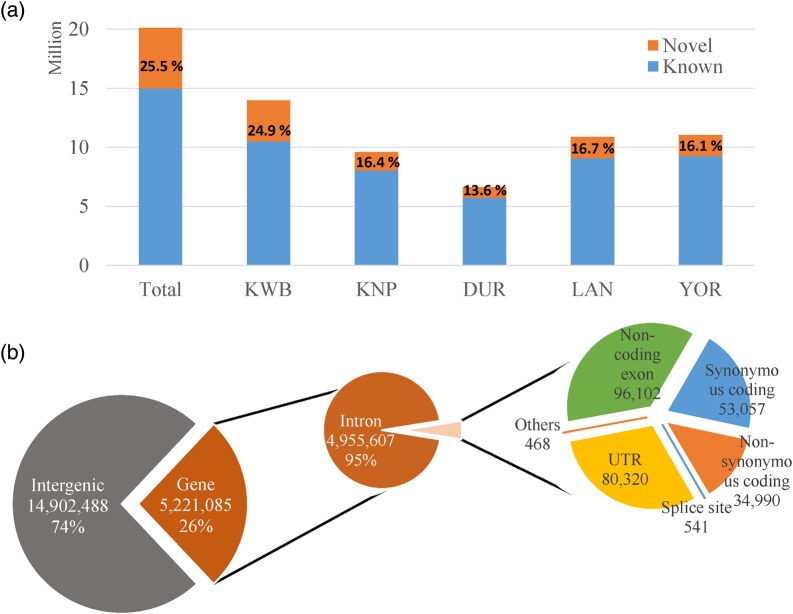
Overview of the identified SNPs. (a) The number of SNPs identified from 55 pigs of the five breeds used in this study (KWB, KNP, DUR, LAN, and YOR). (b) The pie chart showing functional categories of all the detected SNPs. In the functional categories, the ‘Others’ represents the sum of three functional categories including ‘start lost’, ‘stop gained’, and ‘stop lost’. This figure is available in black and white in print and in colour at *DNA Research* online.

We identified a total of 20,123,573 SNPs throughout the genomes from all the 55 sequenced pigs of the five breeds in this study. The number of SNPs in each breed population varied from 6.6 to 14.0 million; the least number of the SNP set was observed in DUR, while the most values were found in KWB (Table 2). This was as expected, because the DUR was the breed used to construct the Sscrofa 10.2 reference assembly, and the KWB is the most genetically distant of the pig breeds in this study.^1,25^ In addition, we do not rule out the possibility that parts of the SNPs could be caused by the within-population variation in this study. Of the total SNPs, ∼25.5% were found to be novel, as assessed by the dbSNP build 137 (Fig. 1a). Correspondingly, KWB showed the highest percentage of novel SNPs (24.9%) compared with the other four breeds (16.4, 13.6, 16.7, and 16.1% for KNP, DUR, LAN, and YOR, respectively). These values indicated that many SNPs remain to be identified by further sequencing efforts, although numerous SNPs have accumulated since the recent completion of the swine sequencing project. The quality of the detected SNPs was examined by calculating the transition-to-transversion ratio (Ti/Tv) for each SNP (Table 2) as well as experimental validation performing a concordance test. The Ti/Tv ratio has been used as an indicator of potential sequencing errors, and it has been empirically approximated to around 2.1 and 2.2 in recent resequencing studies in Humans and cattle, respectively.^26–28^ Following the previous studies, Ti/Tv ratios for most of the pigs are in accordance with the values observed in the Human and cattle, with no outstanding values (KWB: 1.97, KNP: 1.92, DUR: 1.91, LAN: 1.93, YOR: 1.92). Furthermore, we genotyped a part of the same individuals sequenced in this study, using the Illumina PorcineSNP60K BeadChip to perform a concordance test between the SNP panel genotype and SNPs derived from this study. A total of 44,903 SNPs were used for the concordance test to show high concordance rates (98.8–99.4%), which is reliable enough for further investigations (Supplementary Table S1). These results well suggested that most of the SNPs identified in this study were reasonably accurate.

### 3.3. SNP annotation and gene of interest

All the detected SNPs were annotated functionally using Ensembl gene annotation and dbSNP databases, assigning them to 12 functional classes. In agreement with previous studies, most of the SNPs were located in intergenic and intron regions (74.1 and 24.6% respectively), while fewer SNPs (1.3%) resided within exonic region, including exonic, splice sites, and untranslated regions (Fig. 1b and Table 2). Among the genic SNPs, we identified a substantial number (35,458) of non-synonymous SNPs (nsSNPs) in a total of 9,904 genes, which could be associated with traits of interest in pigs (Supplementary Table S2). To prioritize the nsSNPs, all the nsSNPs were further assigned a SIFT score to classify the effects of each nsSNP on phenotypic functions, based on the amino acid conservation.^29^ According to the typical interpretation of the score, a score of ≤0.05 classifies an nsSNP as ‘damaging’ and a score of >0.05 corresponds to ‘tolerant’. There were 6,849 damaging and 27,215 tolerant nsSNPs in our data (Supplementary Table S2). Furthermore, we extracted nsSNP sets that did not overlap among the breeds in this study (breed-specific nsSNP) and identified 126, 26, 11, 10, and 8 for KWB, KNP, DUR, LAN, and YOR, respectively (Supplementary Table S3).

Of the breed-specific nsSNP, we identified several interesting nsSNPs that may be implicated with economically important traits in the pig. For example, a YOR-specific novel nsSNP (SIFT score: 0.03; 6,916,234 bp on chromosome 2) was located in the phospholipase C, beta 3 gene (*PLCB3*). *PLCB3* is involved in the mammalian oocyte at fertilization, spermatozoa activation, and embryonic lethality in sea urchin and mouse studies.^30–32^ Notably, the Yorkshire (YOR) has been widely used as a maternal line breed, particularly for its large litter size; thus, the SNP in *PLCB3* is a candidate SNP to account for genetic variation in the reproduction trait, whose genetic effects have been difficult to predict. Little is known about which genes affect litter size, which has a low heritability and is a highly appreciated trait in the pig industry.^33,34^ A homozygous SNP was found in all 10 KWB individuals sequenced in this study (GG against the A reference allele). Interestingly, the same pattern was observed in the European wild boar samples, while the G allele frequency is around 33% in the Asian wild boars in a recent independent study (Dr Martien Groenen, personal communication). The gene affected by the SNP has homology to Neurexophilin and PC-esterase domain family, member 4 (*NXPE4*). Members of the NXPE family are neuropeptides that potentially signal via alpha-neurexins.^35^ It could be hypothesized that the NXPE4 homolog is involved in neurological mechanisms associated with temperament or fear, whose genetic mechanisms are poorly understood in pigs. Although it is beyond the scope of this study to conclude how each of the genes are affected by the nsSNPs, the many damaging nsSNPs identified provide ample information to identify promising candidates for further studies to dissect genetic mechanisms of diverse traits in pigs.

### 3.4. Phylogenetic and admixture analysis

**Figure 2.**
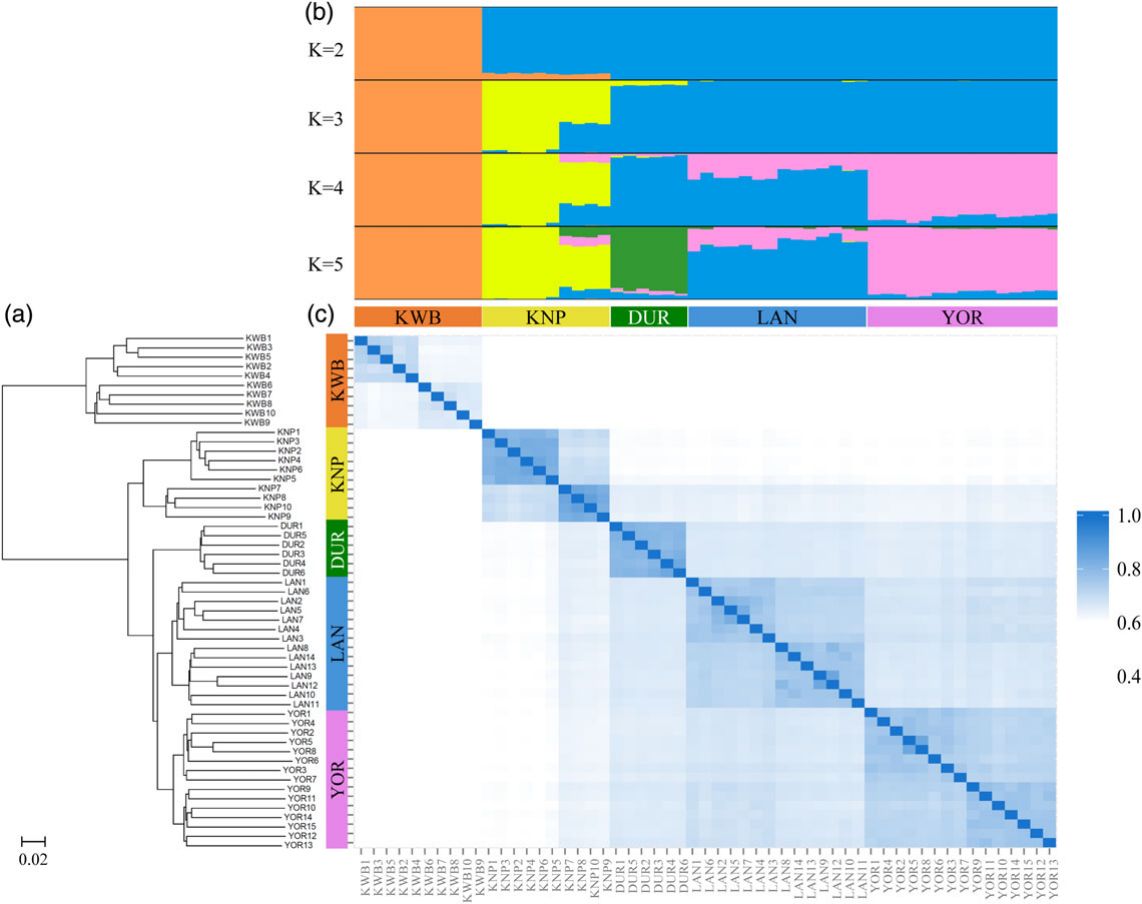
Genetic structure of the 55 pigs of the five pig breeds (KWB, KNP, DUR, LAN, and YOR) in this study. (a) Neighbour-joining phylogenetic tree of all the 55 pigs. (b) Population structure using the STRUCTURE program, which Bayesian cluster all 55 samples from the five breeds. (c) The similarity matrix of the 55 pigs based on the calculated SNP identity. Each of all the 55 pigs was presented in the same order on those three (a–c) plots. This figure is available in black and white in print and in colour at *DNA Research* online.

To examine the population structure and genetic relationships of the five breeds, we carried out multiple analyses, including phylogenetic analysis by neighbour-joining estimation, similarity estimation by comparison of genotypes, and admixture analysis using the STRUCTURE program. All five breeds were consistently separated by those three methods, as expected. The wild boar (KWB) was mostly distinguished from the four domesticated breeds (KNP, DUR, LAN, and YOR), and a clear genetic distance was also observed within the domesticated breeds with the respect to each breed (Fig. 2a). The result was further supported by the similarity matrix, which showed genotyping similarity of <41% between all the KWBs and the domesticated breed individuals. KWB appears to be genetically closer to KNP than any other breed, as shown in both the phylogenetic tree and similarity matrix. The similarity level between KWB and KNP varied from 38.8 to 41%, while that between KWB and the other three breeds was 36.7–38.9%. We observed the maximum value of the genotyping similarity (∼70%) across the domestic breeds, while the minimum values within each breed were >70%, except for the KNP (minimum value = 68.1%). In the STRUCTURE analysis, we found the best-fit value of *K* as 5 to estimate the most likely number of genetic clusters, which corresponded to the number of breeds used in the study. While observing obvious genetic differentiation among the breeds, LAN and YOR have partially shared genetic differentiation signals; YOR shared 4–12.5% of LAN and LAN shared 8.3–31.5% of the YOR. Interestingly, among the 10 KNP individuals, six animals (Chookjin Chamdon, KNP Nos. 1–6) appeared to be genetically distant from the other four KNPs (Jeju native pig, KNP Nos. 7–10) that share other breed populations (11.3–14.6, 12.8–17.3, and 11.4–13.3% for DUR, LAN, and YOR, respectively). The result was found consistently in the phylogenetic tree, similarity matrix, and STRUCTURE analyses in this study. KNPs can be classified as two subgroups with respect to their geographical location in Korea, and they have been bred separately for ∼50 yrs. Despite their same origin until the last century, this result may imply that the Chookjin Chamdon and Jeju native have discernable genetic differences that may be caused by their geographical isolation and different environments. In addition, we did not rule out the possibility that the native breed may have been affected by unrecorded crossbreeding with imported breeds before the systematic management of the native genetic resources.

### 3.5. Selective sweep analysis

**Figure 3.**
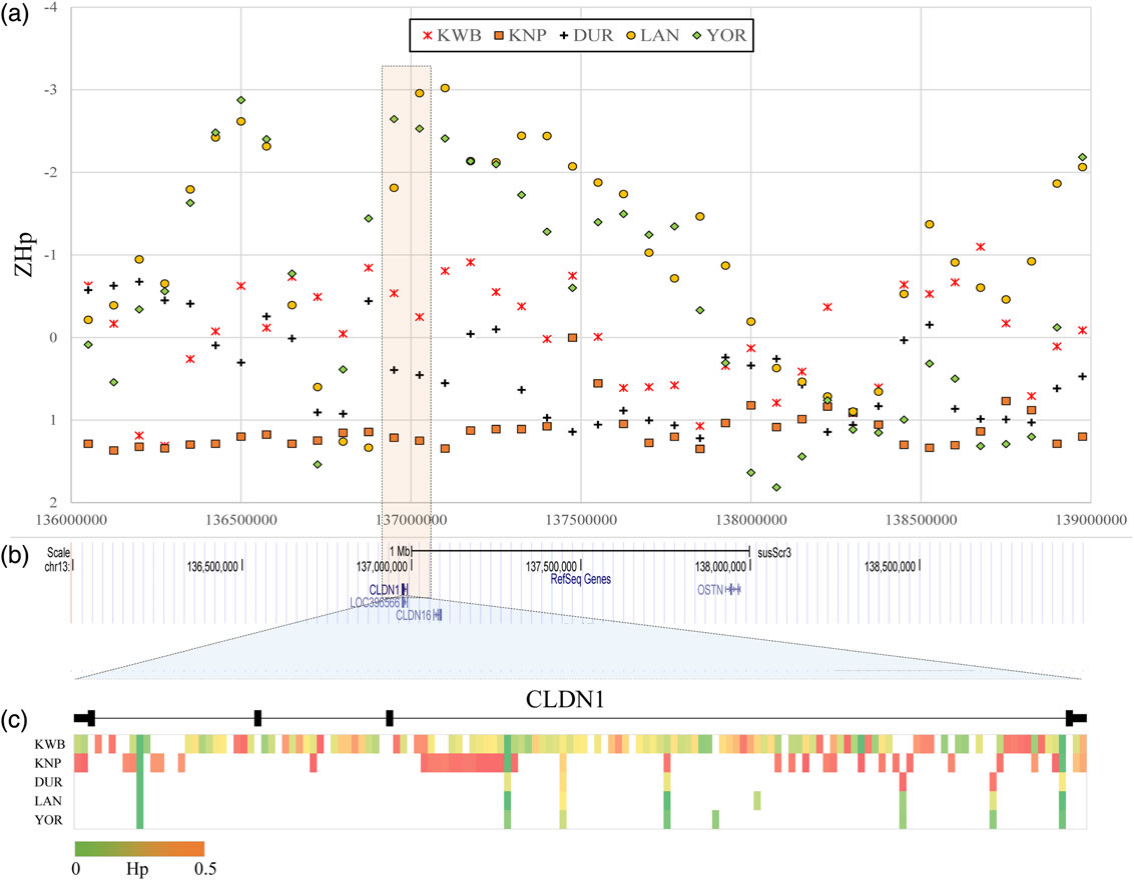
*Z*-scores of pooled heterozygosity (ZHp) overlapping the *CLDN1* gene region. The highlighted orange-coloured box in the dotted line indicates genomic regions including the *CLDN1* gene. (a) ZHp scores at the region of 136–139 Mb on Swine chromosome 13. Each point represents a ZHp score estimated from a 150 kb window. Five different shapes of the points represent each breed, respectively (see the legend for details). (b) The structure of RefSeq genes included in the 136–139 Mb region, which were presented by the UCSC genome browser. (c) Hp score of each SNP detected in the *CLDN1*. Colour gradation was used to show the degree of homozygosity: red (Hp = 0) and green (Hp = 0.5) colours represent the complete homozygosity and heterozygosity, respectively. Namely, high level of homozygosity represents the deepest red colour, while high level of heterozygosity increases green colour intensity. This figure is available in black and white in print and in colour at *DNA Research* online.

The whole genomes were scanned to identify genomic regions with excess homozygosity as indicative of a signature of selection in each breed. By applying 50% overlapping windows of 150 kb in size, 32,651 windows were used in the genome scanning. All the detected SNPs in each breed were used to calculate the ZHp. Altogether, 236, 472, 1,568, 383, and 363 windows were removed that had fewer than 10 SNPs in KWB, KNP, DUR, LAN, and YOR, respectively. In each breed, the ZHp values varied from −4.83 to 2.73, −3.19 to 1.58, −2.57 to 1.45, −3.60 to 2.07, and −3.54 to 1.98 (Supplementary Figure S1), and we observed 1,277, 1,219, 656, 759, and 904 windows with ZHp values lower than −2 for KWB, KNP, DUR, LAN, and YOR, respectively (Supplementary Table S4–S8). An extremely low ZHp score is indicative of a selective sweep; therefore, we further accessed windows having low ZHp scores. Thus, we located several convincing loci that are potentially implicated with the selection applied on each breed. For example, the Claudin-1 (*CLDN1*) gene is located on chromosome 13 (Position: 136,971,662–136,987,446 bp), and windows including the gene (bin#: 1,815 and 1,826) exhibited a putative signature of selection in both LAN (ZHp = −1.81 and −2.96) and YOR (ZHp = −2.64 and −2.53) pigs (Supplementary Table S5 and S6). The *CLDN1* genomic region was further examined by accessing pooled heterozygosity (Hp) calculated from each allele of the 142 SNPs that were identified in the region (Fig. 3c). Given that Hp scores of 0 and 0.5 indicate complete homozygosity and heterozygosity, respectively, three breeds other than the LAN and YOR showed distinctively lower homozygosity at the *CLDN1*. The *CLDN1* is a dominant tight junction protein that plays a role in invasion activity during metastasis, and its biological functions are known to maintain pregnancy and facilitate conceptus attachment.^36,37^ Note that, of the five breeds in this study, both LAN and YOR are outstanding maternal line breeds, with superior reproduction traits. In addition, a recent report showed that the Claudin family members are expressed more abundantly in Yorkshire pigs, as assessed using the porcine Affymetrix-Chip, suggesting that it may be implicated with successful conceptus attachment in this breed.^38^

**Figure 4.**
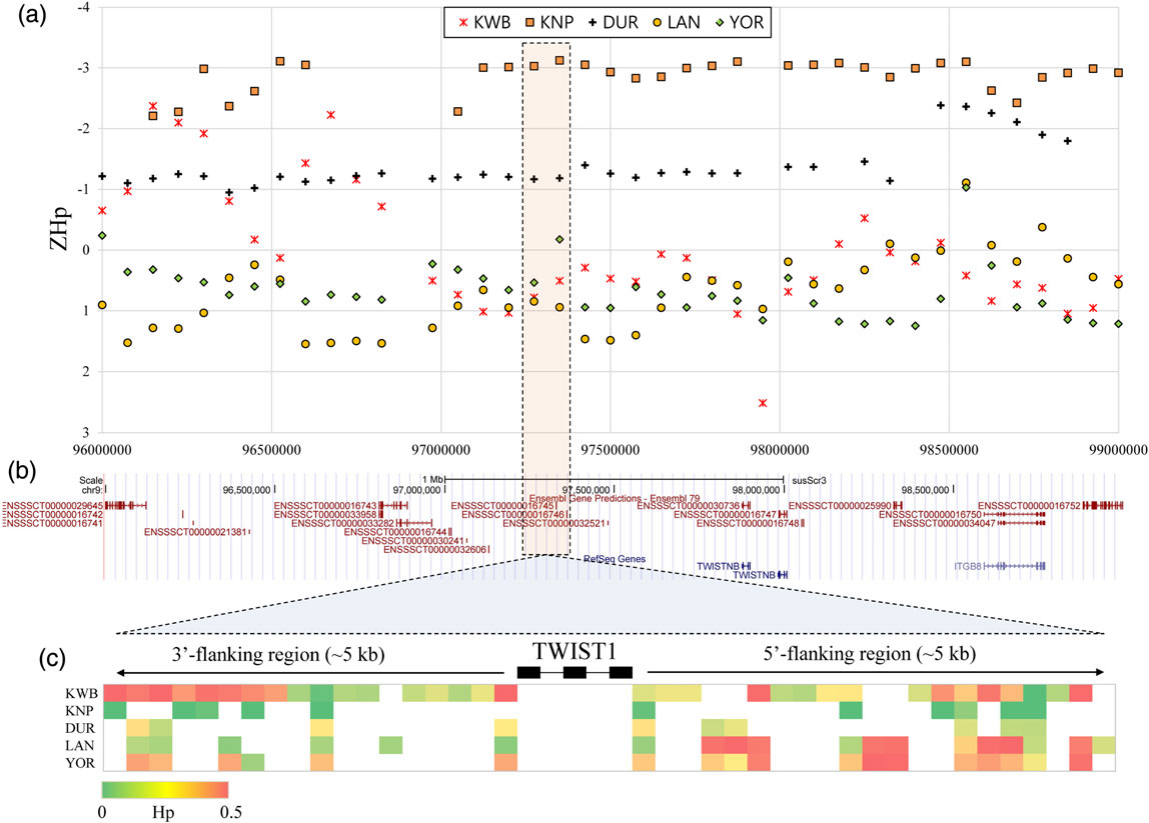
*Z*-scores of pooled heterozygosity (ZHp) overlapping the *TWIST1* gene region. The highlighted orange-coloured box in the dotted line indicates genomic regions including the *TWIST1* gene. (a) ZHp scores at the region of 96–99 Mb on Swine chromosome 9. Each point represents a ZHp score estimated from a 150 kb window. (b) The structure of Ensembl and RefSeq genes included in the 96–99 Mb region, which were presented by the UCSC genome browser. (c) Hp score of each SNP detected in the *TWIST1*. The presented region includes 5 kb flanking regions at both sides of the *TWIST1* gene. This figure is available in black and white in print and in colour at *DNA Research* online.

As another example, we found a signature of selection for a locus on Chromosome 9 (window #1296), which includes the twist family bHLH transcription factor 1 gene (*TWIST1*). The genomic region showed a distinctly lower ZHp of −3.02 in KNP (Supplementary Table S5) compared with the other four breed populations (0.78, −1.17, 0.85, and 0.53 for KWB, DUR, LAN, and YOR, respectively). *TWIST1* is implicated in cell lineage differentiation and is associated with breast cancer and Saethre–Chotzen syndrome in humans.^39,40^ Furthermore, its expression was low in obese subjects and increased after weight loss, suggesting a prospective role in obesity.^41,42^ Despite its lower growth rate and litter size, KNP is known to have the higher intramuscular fat content (marbling) than typical imported breeds. The marbling is an important palatability factor appealing to many Korean pork consumers; however, limited numbers of genes have been identified to account for genetic variations in the trait in KNP. These examples show the potential of our analysis to identify candidate genes to dissect genetic mechanisms implicated in economically important traits in diverse pig breeds (Fig. 4).

## 4. Conclusions

Here, we present extensive whole-genome resequencing analyses for 55 pigs of five breeds: Korean wild boar, Korean native, Duroc, Landrace, and Yorkshire. This study had two main findings. First, we identified a significant number of SNPs across the genomes (∼20.1 million), of which substantial numbers were novel. Furthermore, we located numerous nsSNPs using a deep annotation process, which could be candidate genetic markers for predicting genetic variations in traits of interest. Second, multiple methodologies were applied to the detected SNPs to dissect genomic features of the pig breeds, revealing obvious signals of genetic differentiation among the breeds. Furthermore, the whole genomes were scanned to detect signatures of selection by accessing excess homozygosity throughout the genome. We observed many higher homozygosity regions, which are unevenly distributed across the genome. The results allowed us to retrieve several interesting genomic regions that may be associated with economically important traits in pigs.

## Authors’ contribution

J.W.C., W.H.C., and K.T.L. wrote the article. N.K. and T.H.K. conceived and designed the experiments. J.W.C., W.H.C., K.T.L., S.H.L., W.L., D.L., Y.G.L., J.K., N.K., and T.H.K. carried out the analysis of the genome sequences. K.T.L., E.S.C., and T.H.K. performed the experiments.

## Supplementary data

Supplementary data are available at www.dnaresearch.oxfordjournals.org

## Funding

This work was supported by 2-7-10 Agenda Research (PJ00670701) from the National Institute of Animal Science; a grant (PJ008068) from the Next-generation BioGreen 21 Program, Rural Development Administration, Republic of Korea; and the 2014 Post-doctoral Fellowship Program of the Rural Development Administration, Republic of Korea. Funding to pay the Open Access publication charges for this article was provided by the National Institute of Animal Science.

## Supplementary Material

Supplementary Data
