# Supplementary material for: Whole-genome resequencing analyses of five pig breeds, including Korean wild and native, and three European origin breeds
Source: DNA Res. 2015 Jun 27;22(4):259–67. doi: 10.1093/dnares/dsv011 (PMC4535618; doi:10.1093/dnares/dsv011)
Supplement: Supplementary Data [file supp_22_4_259__index.html]

Whole-genome resequencing analyses of five pig breeds, including Korean wild and native, and three European origin breeds — Whole-genome resequencing analyses of five pig breeds, including Korean wild and native, and three European origin breeds — Supplementary Data 

# Whole-genome resequencing analyses of five pig breeds, including Korean wild and native, and three European origin breeds

## Supplementary Data

Supplementary Data

- Supplementary Figure 1 - ppt file
- Supplementary Table 1 - xlsx file
- Supplementary Table 2 - xlsx file
- Supplementary Table 3 - xlsx file
- Supplementary Table 4 - xlsx file
- Supplementary Table 5 - xlsx file
- Supplementary Table 6 - xlsx file
- Supplementary Table 7 - xlsx file
- Supplementary Table 8 - xlsx file
