# Supplementary material for: Whole-genome resequencing analyses of five pig breeds, including Korean wild and native, and three European origin breeds
Source: DNA Res. 2015 Jun 27;22(4):259–67. doi: 10.1093/dnares/dsv011 (PMC4535618; doi:10.1093/dnares/dsv011)
Supplement: Supplementary Data [file supp_dsv011_dsv011supp_fig1.ppt]

## Slide 1
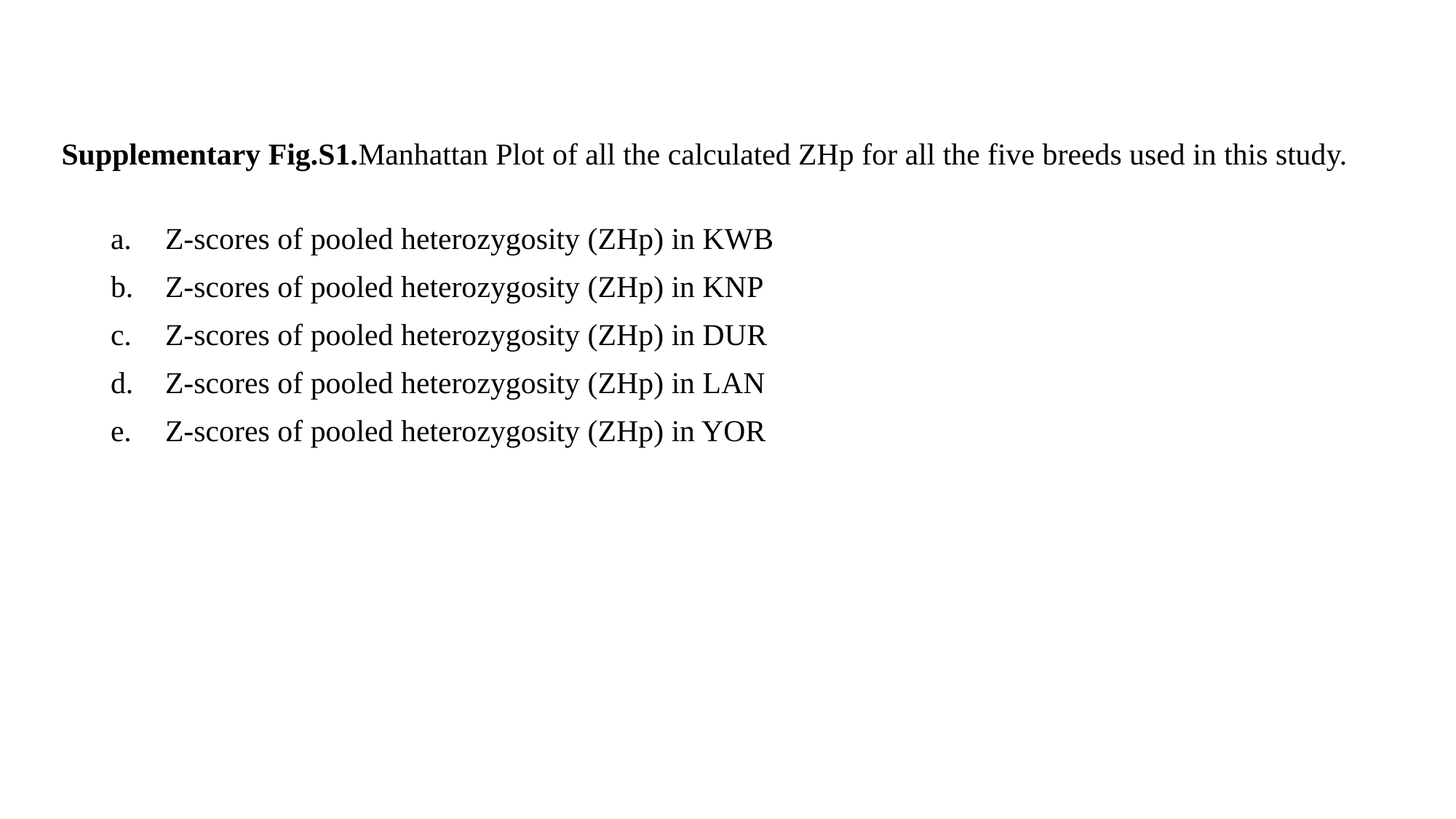

# Supplementary Fig.S1.Manhattan Plot of all the calculated ZHp for all the five breeds used in this study.
Z-scores of pooled heterozygosity (ZHp) in KWB
Z-scores of pooled heterozygosity (ZHp) in KNP
Z-scores of pooled heterozygosity (ZHp) in DUR
Z-scores of pooled heterozygosity (ZHp) in LAN
Z-scores of pooled heterozygosity (ZHp) in YOR

## Slide 2
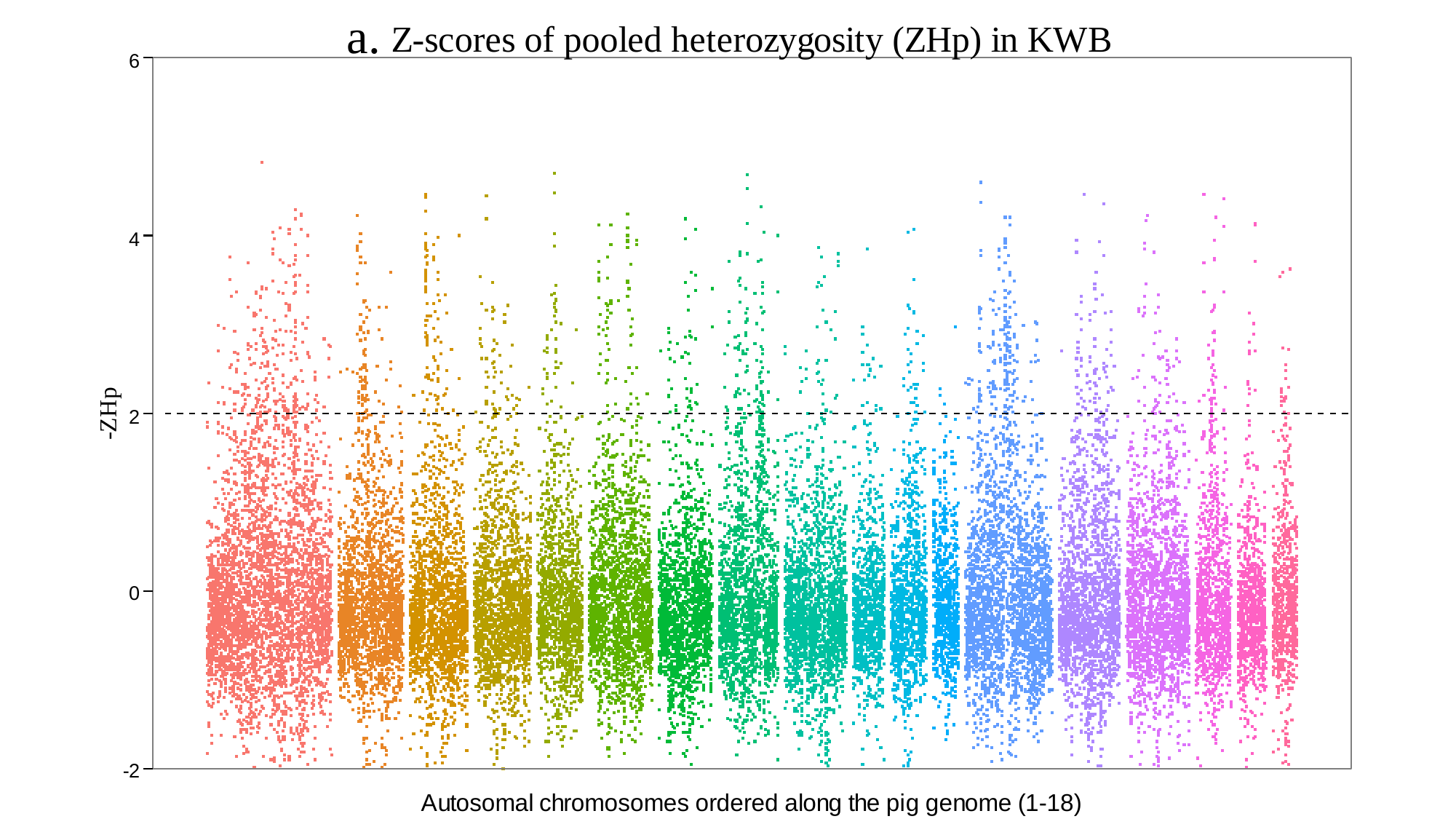

a.
#

## Slide 3
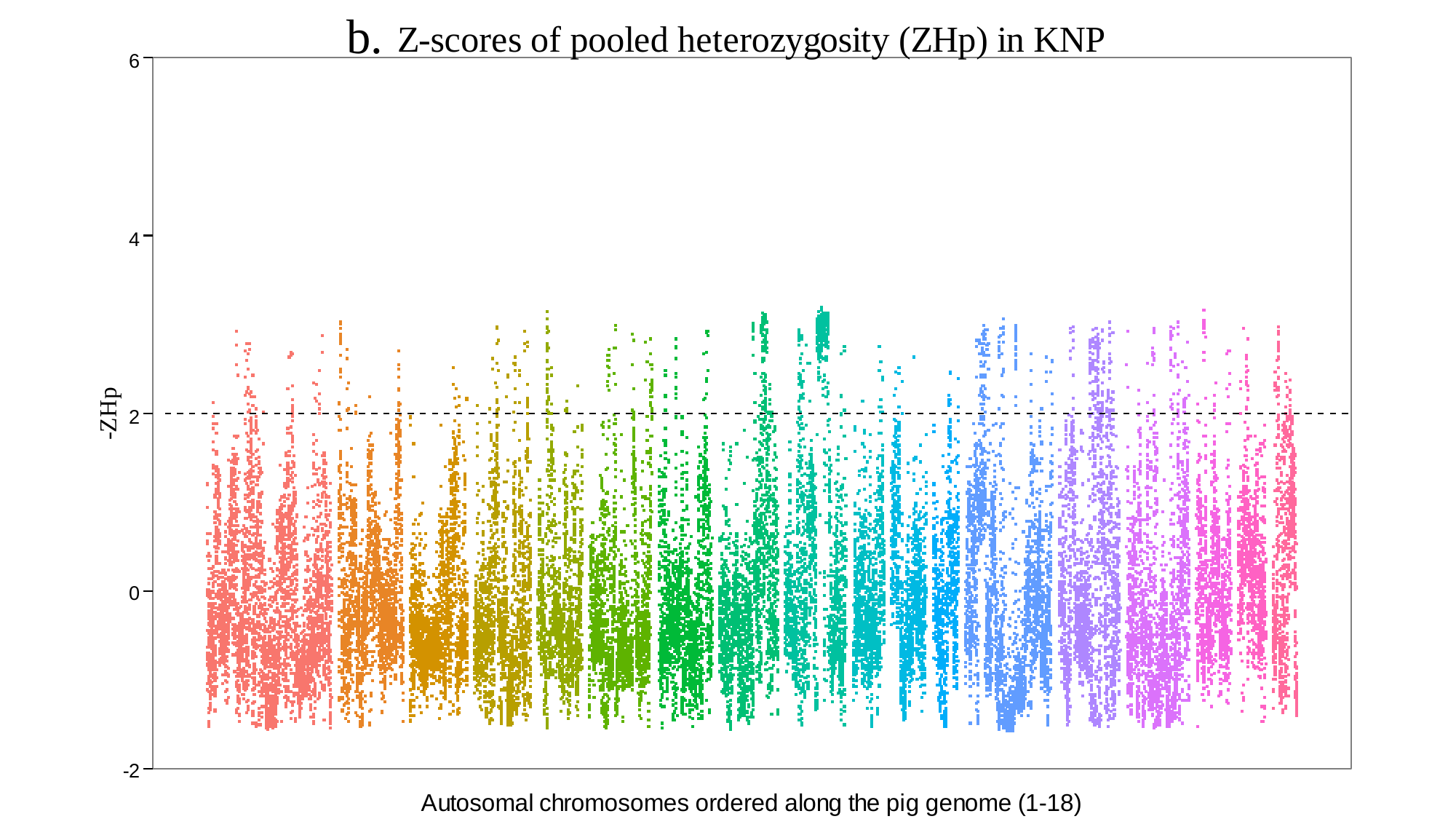

b.

## Slide 4
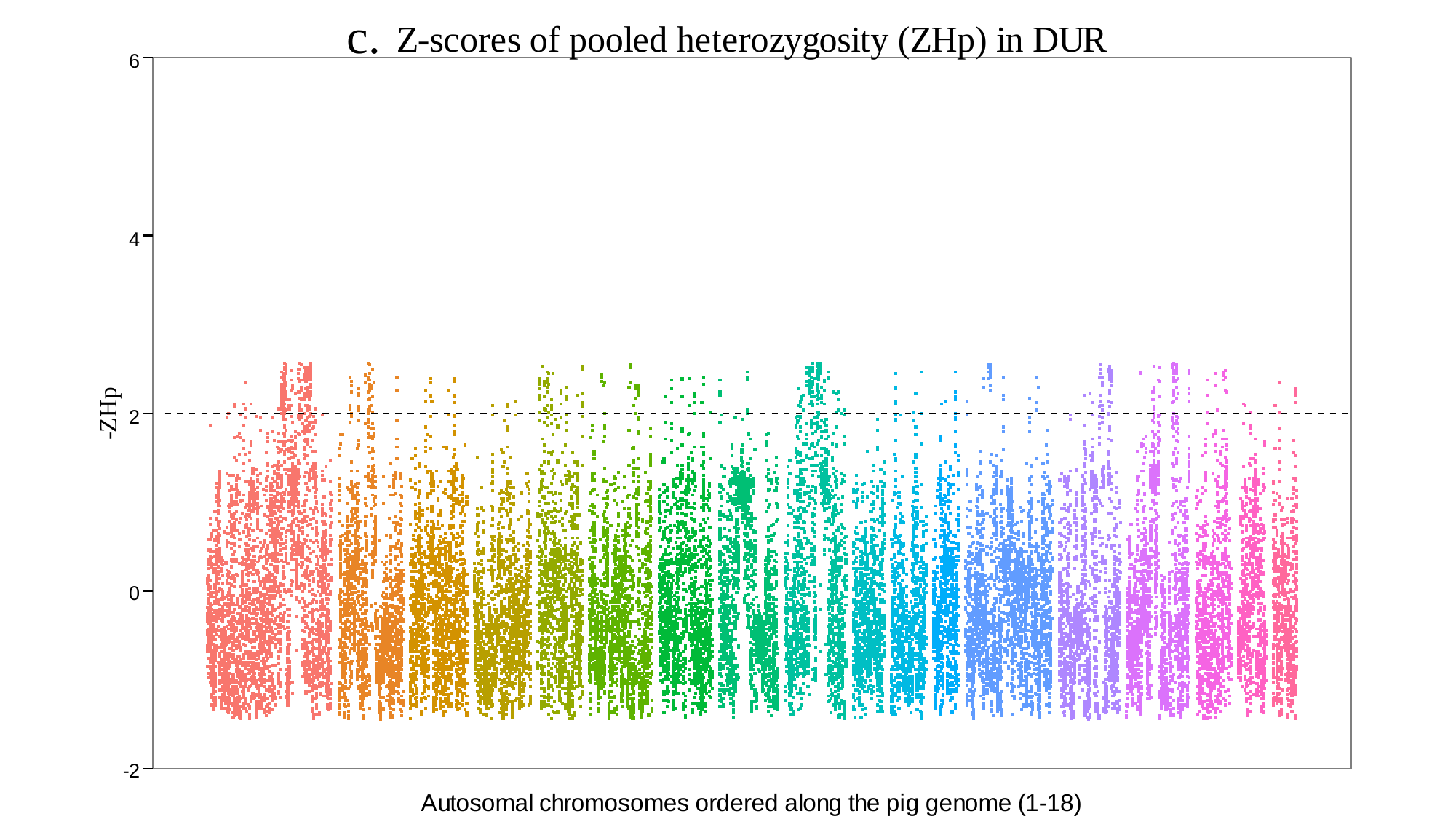

c.

## Slide 5
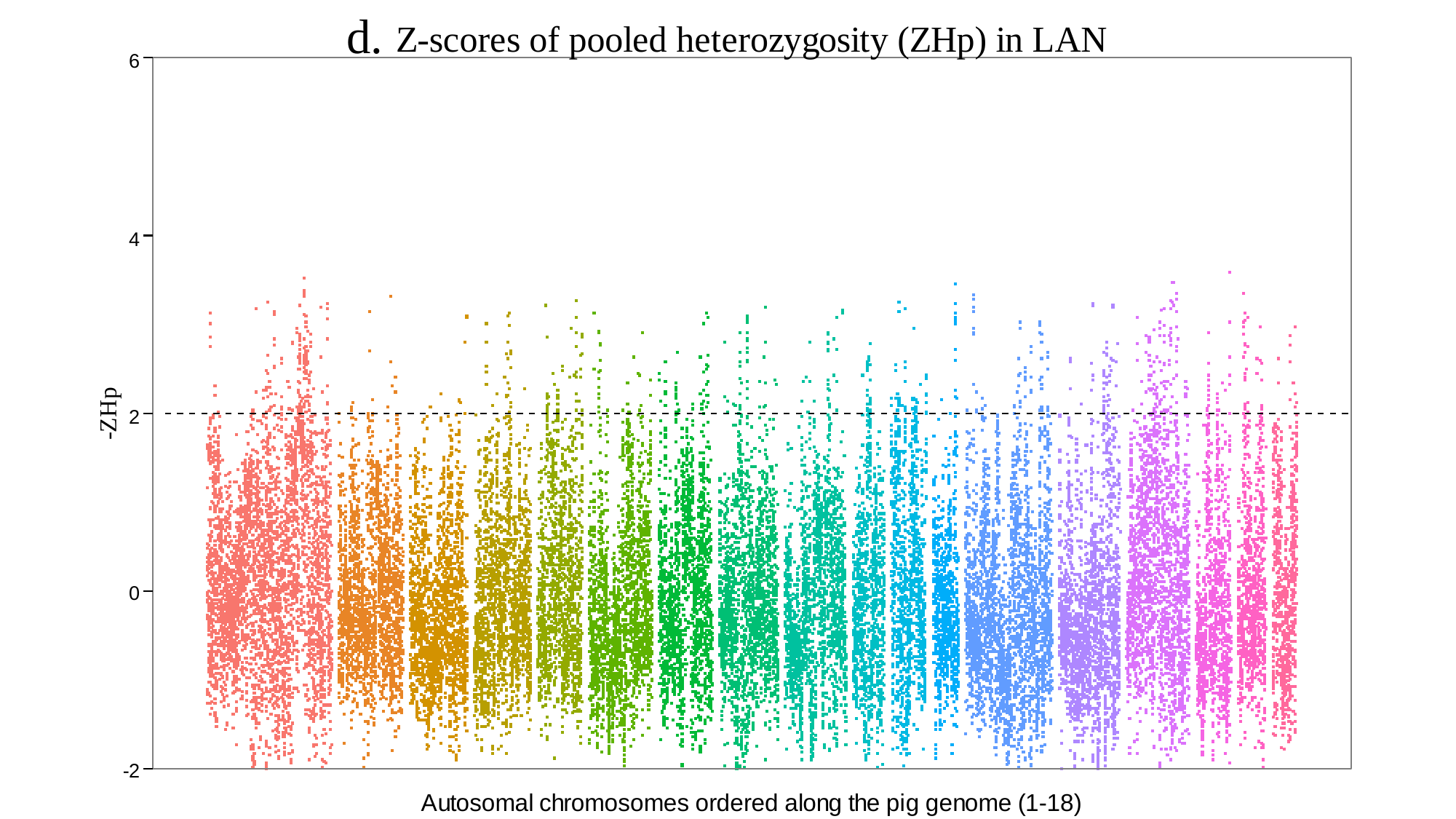

d.

## Slide 6
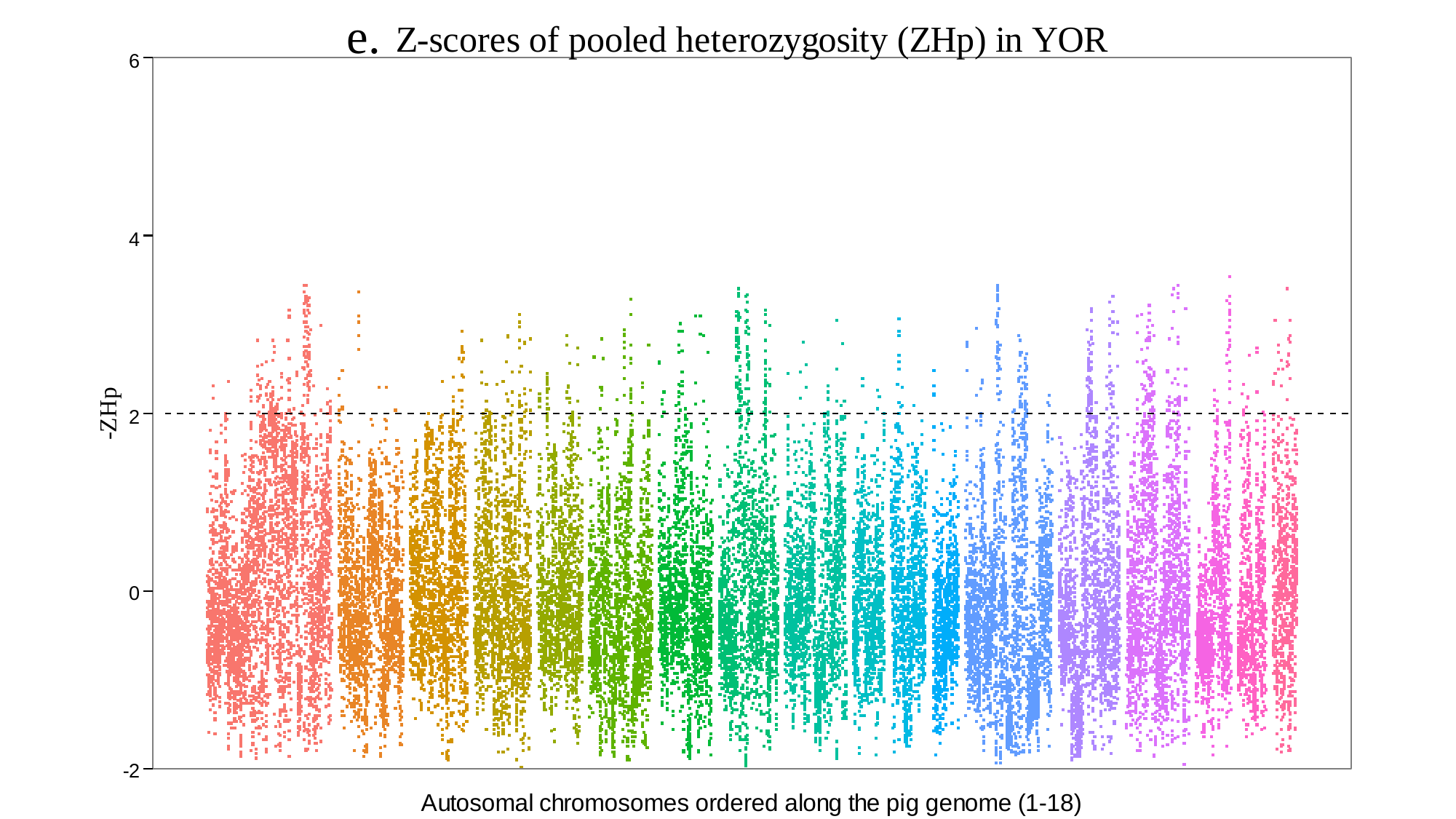

e.
